# *Helicobacter pylori* and Gastric Cancer: Adaptive Cellular Mechanisms Involved in Disease Progression

**DOI:** 10.3389/fmicb.2018.00005

**Published:** 2018-01-22

**Authors:** Paula Díaz, Manuel Valenzuela Valderrama, Jimena Bravo, Andrew F. G. Quest

**Affiliations:** ^1^Cellular Communication Laboratory, Instituto de Ciencias Biomédicas, Facultad de Medicina, Universidad de Chile, Santiago, Chile; ^2^Advanced Center for Chronic Diseases, Facultad de Medicina, Universidad de Chile, Santiago, Chile; ^3^Center for Molecular Studies of the Cell, Facultad de Medicina, Universidad de Chile, Santiago, Chile; ^4^Instituto de Investigación e Innovación en Salud, Facultad de Ciencias de la Salud, Universidad Central de Chile, Santiago, Chile

**Keywords:** *Helicobacter pylori*, precancerous lesion, gastric cancer, endoplasmic reticulum stress, autophagy, inflammation, oxidative stress

## Abstract

*Helicobacter pylori* (*H. pylori*) infection is the major risk factor associated with the development of gastric cancer. The transition from normal mucosa to non-atrophic gastritis, triggered primarily by *H. pylori* infection, initiates precancerous lesions which may then progress to atrophic gastritis and intestinal metaplasia. Further progression to dysplasia and gastric cancer is generally believed to be attributable to processes that no longer require the presence of *H. pylori*. The responses that develop upon *H. pylori* infection are directly mediated through the action of bacterial virulence factors, which drive the initial events associated with transformation of infected gastric cells. Besides genetic and to date poorly defined environmental factors, alterations in gastric cell stress-adaptive mechanisms due to *H. pylori* appear to be crucial during chronic infection and gastric disease progression. Firstly, *H. pylori* infection promotes gastric cell death and reduced epithelial cell turnover in the majority of infected cells, resulting in primary tissue lesions associated with an initial inflammatory response. However, in the remaining gastric cell population, adaptive responses are induced that increase cell survival and proliferation, resulting in the acquisition of potentially malignant characteristics that may lead to precancerous gastric lesions. Thus, deregulation of these intrinsic survival-related responses to *H. pylori* infection emerge as potential culprits in promoting disease progression. This review will highlight the most relevant cellular adaptive mechanisms triggered upon *H. pylori* infection, including endoplasmic reticulum stress and the unfolded protein response, autophagy, oxidative stress, and inflammation, together with a subsequent discussion on how these factors may participate in the progression of a precancerous lesion. Finally, this review will shed light on how these mechanisms may be exploited as pharmacological targets, in the perspective of opening up new therapeutic alternatives for non-invasive risk control in gastric cancer.

## Introduction

Worldwide, gastric cancer (GC) is the fifth most commonly diagnosed malignancy, and the third leading cause of cancer-related deaths per year (IARC, 2014; Lordick et al., 2014). *Helicobacter pylori* (*H. pylori*) is a Gram-negative, flagellated, microaerophilic bacterium that grows in close association with the lining of the stomach and the presence of this infection is identified as the major known risk factor associated with the development of GC (Suerbaum and Michetti, 2002). Epidemiological studies report that 2–3% of *H. pylori*-infected individuals eventually develop GC (Herrera and Parsonnet, 2009).

Generally, GC is viewed as the consequence of a multifactorial process, involving the host responses, bacterial virulence, diet, and other environmental factors (Valenzuela et al., 2015). The intestinal-type adenocarcinoma is the most frequent GC diagnosed (Hu et al., 2012). GC development is a multistep process initiated by the transition of normal mucosa to chronic superficial gastritis (non-atrophic gastritis), triggered primarily by *H. pylori* infection. Gastritis may progress to atrophic gastritis, then intestinal metaplasia, and finally to dysplasia and adenocarcinoma (Correa, 1992; Correa and Houghton, 2007). Prospective studies have shown that antibiotic-mediated eradication of *H. pylori* significantly reduces the incidence of precancerous lesions and thus highlights the role of *H. pylori* infection in early stages of gastric carcinogenesis (Mera et al., 2005). Indeed, removal of the bacterium with antibiotics can contribute to regression of atrophic gastritis, however this course of action is no longer effective once the disease has progressed to the stage of intestinal metaplasia (Massarrat et al., 2012). In accordance, eradication of *H. pylori* in patients with metaplasia and dysplasia does not reduce the risk of GC (Chen et al., 2016). These data suggest that the transition from atrophic gastritis to intestinal metaplasia is a crucial step in the progression towards GC and underscore the role of *H. pylori* in the initiation of the multistep cascade leading to precancerous lesions.

The inflammatory response that develops upon *H. pylori* infection is directly mediated through the action of a variety of bacterial virulence factors on host gastric epithelial cells (Peek and Crabtree, 2006). Pathogenicity of *H. pylori* is attributed to bacterial factors including, but not limited to, urease, vacuolating cytotoxin A (VacA), cag pathogenicity island, cytotoxin-associated gene A (CagA), peptidoglycan outer membrane proteins (e.g., BabA, SabA, OipA), and γ-glutamyl transpeptidase (GGT) (Polk and Peek, 2010; Valenzuela et al., 2013).

**Figure 1:**
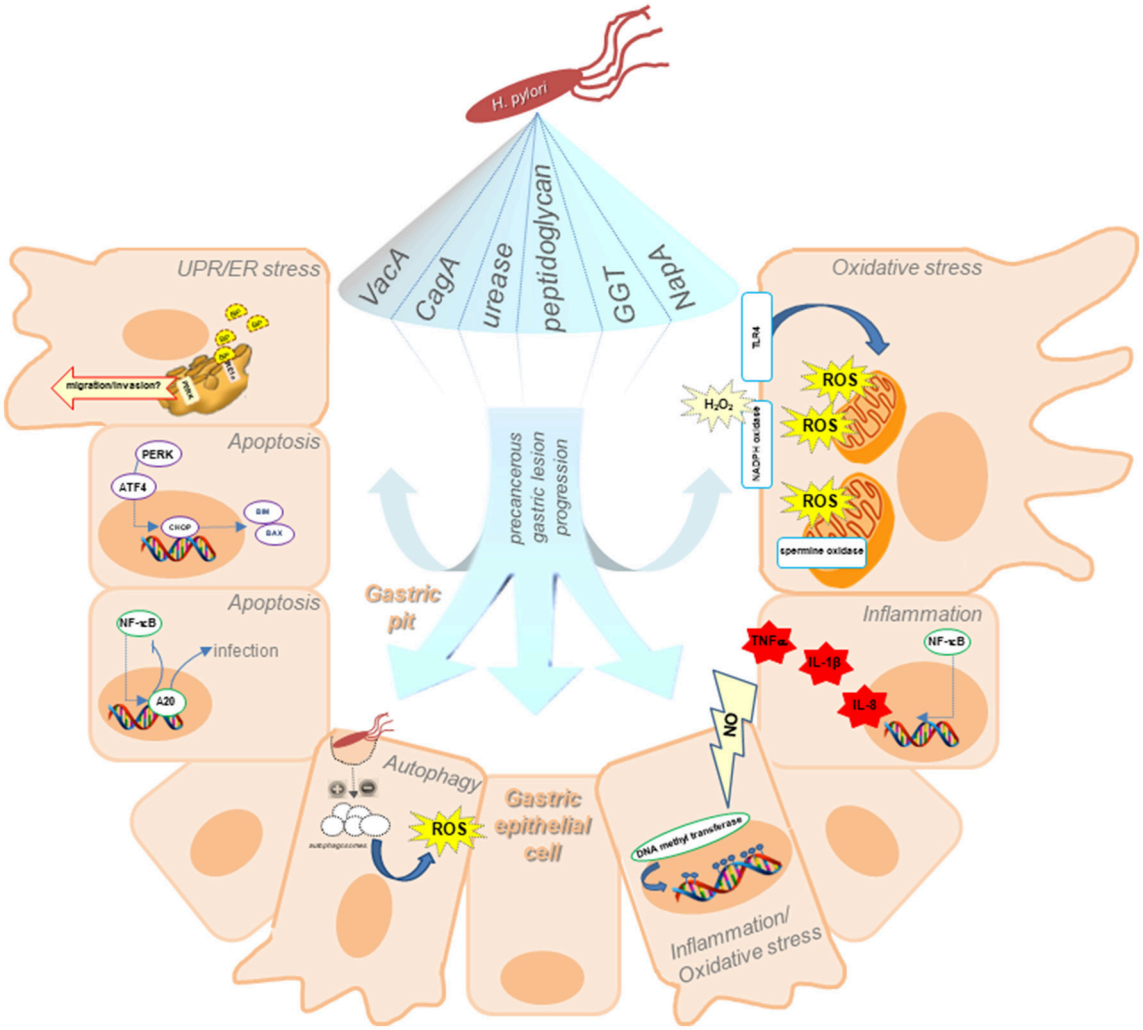
Schematic illustration of our current understanding of adaptive cellular mechanisms triggered upon *H. pylori* infection, including ER stress and the UPR, autophagy, oxidative stress, and inflammation, indicating how they may participate in precancerous lesion progression. Responses in host gastric epithelial cells located in the gastric pits triggered upon *H. pylori* infection are attributable to the action of bacterial virulence factors. ER stress associated with *H. pylori* infection, leads to an increase in BiP, suggesting that *H. pylori*-induction of ER stress is relevant in early stages of GC precancerous lesions. The ER stress sensor PERK may also facilitate tumor development by increasing the migratory and invasive potential of gastric cells. Unresolved ER stress results in apoptosis. ER-stress induced apoptosis is mediated by the transcription of CHOP, leading to expression of the pro-apoptotic proteins Bim and Bax. Moreover, *H. pylori* benefits from NF-κB activation and negatively regulates apoptosis via A20 deubiquitinylase activity, thereby promoting persistence of the infection. Inhibition (or activation) of autophagy, resulting in accumulation of autophagosomes within the cell at the beginning of the precancerous cascade are depicted as increasing ROS production leading to persistent oxidative stress, which in turn promotes the acquisition of characteristics, favoring invasion and metastasis. Long-term inflammation of the gastric mucosa generates significant amounts of nitric oxide (NO), which alters the transcriptional regulation in gastric cells by increasing DNA methyl transferase activity. The resulting hypermethylation of gene promoter sequences leads to epigenetic changes in gene expression. Additionally, NF-κB target genes include those representing polymorphisms associated with an increased risk for GC in patients, such as TNFα, IL-1β, and IL-8. Gastric cells produce ROS in response to *H. pylori* infection by inducing pro-oxidant activities, such as the host spermine oxidase, NADPH oxidase or generating ROS from mitochondria following activation of TLR4 signaling.

Besides genetic and environmental factors, alterations in gastric cell adaptive mechanisms due to *H. pylori* provoked stress appear to be crucial during chronic infection and gastric disorders. Initially, the *H. pylori*-induced effects observed during gastritis (and throughout the beginning of intestinal metaplasia) are an increase in apoptosis and a reduction in cell turnover in the majority of infected epithelial cells, thus resulting in atrophy, phenotypic changes, and the development of primary tissue lesions associated with the initial inflammatory response (Polk and Peek, 2010). However, in the remaining gastric cell population, adaptive responses are induced that increase cell survival and proliferation, and thus the acquisition of potentially malignant characteristics that allow the progression of gastric precancerous lesions, invasion, and metastasis. As summarized in Figure 1, deregulation of intrinsic survival-related responses to *H. pylori* infection may emerge as potential culprits in favoring disease progression.

## Endoplasmic reticulum stress and the unfolded protein response are associated with the pathogenesis of *h. pylori*-induced gastric tumourigenesis

Protein folding stress at the endoplasmic reticulum (ER) is involved in the pathogenesis of a variety of human diseases (Hetz, 2012). The accumulation of unfolded and/or misfolded proteins within the ER induces ER stress, which can be resolved by an adaptive mechanism termed the unfolded protein response (UPR) (Hetz, 2012).

Activation of ER stress was found to be significantly associated with *H. pylori*-positive GC (92%) (Baird et al., 2013). In intestinal metaplasia, levels of key modulators of the UPR activated by ER stress such as HSPA5 (encoding for binding immunoglobulin protein; BiP), C/EBP-homologous protein [CHOP; positively controlled by the protein kinase R (PKR)-like ER kinase (PERK) pathway], and XBP1 (downstream of inositol-requiring enzyme-1 alpha; IRE1-α), were reportedly higher in *H. pylori*-positive subjects compared to earlier precancerous stages (i.e., non-atrophic and atrophic gastritis) (Baird et al., 2013). This suggests that *H. pylori*-induction of ER stress may play a significant role in the early stages of precancerous lesion formation.

Above a certain threshold, unresolved ER stress results in apoptosis and evasion of apoptotic cell death is recognized as one of the hallmarks of cancer cells (Hanahan and Weinberg, 2011). Thus, ER stress-induced cell death in the mucosa after *H. pylori* infection may favor the progression from precancerous lesions into GC. ER-stress induced apoptotic cell death is mediated by the transcription of CHOP (Tabas and Ron, 2011). In line with this, it was reported that CHOP is transcriptionally upregulated following incubation of gastric cells with *H. pylori* bacterial virulence factor VacA (Akazawa et al., 2013). Moreover, silencing of the PERK gene (EIF2AK3) attenuated VacA-mediated phosphorylation of eukaryotic initiation factor-2 alpha (eIF2α), expression of BH3-only protein Bim and Bax, as well as cell death induced by VacA (Akazawa et al., 2013), indicating that ER stress may lead to apoptotic cell death during VacA-induced toxicity (Akazawa et al., 2013). In line with this hypothesis, it has been recently reported that *H. pylori* benefits from NF-κB activation and negatively regulates apoptotic cell death via a deubiquitinylase activity, thereby promoting persistence of the infection (Lim et al., 2017).

Furthermore, besides epithelial cells, dendritic cell alteration is also observed in the *H. pylori* infected gastric mucosa. Treatment of dendritic cells with purified *H. pylori* VacA was shown to induce translocation of cytoplasmic Bax and cytochrome c release from mitochondria and thus bring about apoptosis (Kim et al., 2015). Indeed, suppression of ER stress appears to result in a significant inhibition of the VacA-induced apoptotic cell death in dendritic cells, suggesting that ER stress is also critical for regulation of dendritic cell apoptosis in response to VacA stimulation (Kim et al., 2015).

The role of the ER stress sensor PERK in tumor development remains controversial (Ranganathan et al., 2008). In agreement with the classical role of PERK in the UPR, some studies suggest that PERK activation inhibits tumor cell proliferation leading to apoptosis (Akazawa et al., 2013; Huber et al., 2013; Martín-Pérez et al., 2014). However, other reports have shown that PERK activation facilitates tumor development by promoting tumor cell survival and enhancing angiogenesis (Atkins et al., 2013; Mujcic et al., 2013). Consistent with this latter role, PERK promotes tumourigenesis (Bobrovnikova-Marjon et al., 2010) and PERK-deficient cells display a reduced ability to form solid tumors in nude mice (Spiotto et al., 2010). More recently, a new role for PERK signaling in promoting cancer cell migration and invasion was proposed based on experiments evaluating the effects of moderate PERK activation on medulloblastoma cell migration and invasion (Jamison et al., 2015). Overall, these observations support the idea that, besides its classical role in UPR and apoptosis, following *H. pylori* infection, PERK expression also promotes human gastric cell migration and invasion and thus the acquisition of an aggressive phenotype.

## *H. pylori*-mediated autophagy favors the progression of gastric cancer precursor lesions

The link between ER stress and autophagy is well established, and in the context of *H. pylori*, it has been demonstrated that the secreted antigen HP0175 can regulate PERK, which in turn activates the transcription of ATF4 and CHOP leading to the induction of autophagy in gastric epithelial cells (Halder et al., 2015).

Autophagy is a conserved mechanism by which the eukaryotic cell seeks to maintain cellular homeostasis; it is also induced as a survival response upon exposure to various stress stimuli, including deprivation of nutrients and infection by microbial pathogens (He and Klionsky, 2009). Briefly, cellular components destined for degradation are sequestered within the autophagosome that fuses with the lysosome (autolysosome), where the cargo is degraded by lysosomal enzymes and recycled back to the cell (Russell et al., 2014).

More recently, autophagy has also come to be recognized as an important intracellular defense mechanism that challenges pathogenic bacteria after their internalization by the host cell. Several types of pathogens have developed different strategies to either escape from lysosomal degradation, or control and modulate autophagy to their own benefit (Lerena et al., 2010). In the context of *H. pylori* infection, Terebiznik et al. (2009) reported that infection of gastric epithelial cells with *H. pylori* for short periods (6 h) induced autophagy in a manner that was independent of the virulence factors CagA and urease, but dependent on VacA and the latter factor alone was sufficient to induce this effect (Terebiznik et al., 2009). This suggested that autophagy acts as a mechanism by which infected cells limit toxin-induced cellular damage and thus favor cell survival. In a subsequent study, Raju et al. (2012) showed that prolonged exposure to VacA (24 h) disrupted the antiphagocytic pathway and defective autophagosomes were reported to accumulate within the cells (Raju et al., 2012). Moreover, these authors showed that p62, a selective substrate for autophagy-mediated degradation, increased in gastric biopsies of patients infected with the toxic s1m1 VacA strain in comparison with those infected with a nontoxic strain (VacA ^s2m2^). Additionally, it was reported that the effect of *H. pylori* in the regulation of autophagy was linked to changes in the expression of autophagy-related genes in gastric cells and macrophages (Castaño-Rodríguez et al., 2015). In line with this, silencing by methylation of the microtubule-associated protein 1 light chain 3 variant 1 (MAP1LC3Av1), an important protein in the autophagy process, was observed in gastric tissue infected with *H. pylori* (Muhammad et al., 2017). The available literature suggests that *H. pylori* modulates autophagy in the host and points toward VacA as a possible mediator; however, the reported results provide contradictory mechanisms concerning how precisely VacA might regulate this process. In addition, although the effect of virulence factor CagA in autophagy was initially discarded by Terebiznik et al. (2009), this factor has recently been identified as a negative regulator of autophagy in gastric mucosa of patients and AGS cells infected with a non-functional VacA strain (VacA ^s1m2^) (Li et al., 2017). Consequently, CagA might cause the inhibition of autophagy through the c-Met-PI3K/Akt-mTOR signaling pathway. Moreover, these authors also found higher expression of p62 in gastric biopsies of patients infected with CagA+ strains in comparison with those infected with CagA− strains (Li et al., 2017). Considering the latter observation, the differences found in the literature can be explained in part by the use of different cell lines and bacterial strains, however, the possibility that additional *H. pylori* virulence factors are likely to be involved in the regulation of autophagy in gastric cells merits further consideration. Thus, autophagy acts as a quality control system in early stages of cancer, and one may infer that the inhibition of this process at the beginning of the precancerous cascade should increase reactive oxygen species (ROS) production leading to persistent oxidative stress, which in turn causes selective pressure for the acquisition of characteristics, such as increased growth, invasion, and metastasis (Azad et al., 2009). This would, in the case of GC, favor the progression of precursor lesions. In summary, a strong connection between *H. pylori* infection and autophagy is emerging that may prove relevant to GC progression.

## *H. pylori*-associated inflammation

Chronic inflammation contributes to the pathogenesis of several types of cancer (Fernandes et al., 2015) and is particularly relevant in the case of *H. pylori*-associated GC (Valenzuela et al., 2015). *H. pylori* infection is invariably associated with gastritis; however, only a minor proportion of individuals progress through the pre-neoplastic cascade (Peek and Blaser, 2002). Particularly, the interplay between host responses and bacterial virulence factors seems most relevant in determining the extent of inflammation and the clinical outcome (Chmiela et al., 2017), which in turn may be favored by a permissive environment.

Beyond representing an innate response to pathogens, long-term inflammation of the gastric mucosa generates significant amounts of the diffusible molecule nitric oxide (Nam et al., 2004), which contributes not only to the damage of nucleotide bases in host DNA, but also alters the transcriptional regulation in gastric cells by increasing DNA methyl transferase activity (Huang et al., 2012). In doing so, epigenetic changes involving the hypermethylation of DNA sequences, particularly in tumor suppressor genes (coding and non-coding) are observed in the gastric mucosa of infected individuals (Kaise et al., 2008; Valenzuela et al., 2015). Accordingly, the promoter region of the tumor suppressor E-Cadherin is frequently hypermethylated in adult patients infected with *H. pylori* (Perri et al., 2007). Also, the demethylating agent 5-azacytidine has been demonstrated to reduce GC incidence in the Mongolian gerbil model of infection (Niwa et al., 2013). Additionally, chronic exposition to inflammatory cytokines is associated with deregulation of the Hedgehog/GLI signaling pathway, which is known to be involved in the maintenance of gastric physiology (Wessler et al., 2017). In doing so, chief cells are reprogrammed toward a metaplastic phenotype, which together with the dysregulated differentiation of infiltrating gastric stem cells (e.g., bone marrow-derived cells), contributes to the so-called mosaicism in GC (Zhang et al., 2016; Leushacke et al., 2017; Mills and Goldenring, 2017).

T-cell responses are essential for the generation of the inflammatory response following *H. pylori* infection and gastric carcinogenesis as demonstrated in animal models of infection (Kandulski et al., 2010). Notably, *H. pylori* has developed mechanisms to persist in the gastric niche suppressing inflammation at sites of colonization (Every et al., 2011). Accordingly, some virulence factors have been shown to control the extent of inflammatory responses, not only by inhibiting T-cell activation, but also by reducing phagocytosis or by promoting evasion of toll-like receptor (TLR) recognition, as well as by inducing tolerogenic effects in dendritic cells (Mejías-Luque and Gerhard, 2017). Well documented in this context are the actions of bacterial virulence factors, such as the secreted vacuolating toxin VacA and GGT, as well as the oncogenic cytotoxin CagA. Present in more virulent and carcinogenic strains, CagA is injected via the type IV secretion system encoded in the bacterial pathogenicity island (CagPAI) (Miyaji et al., 2000; Cha et al., 2010; Mejías-Luque and Gerhard, 2017). GGT, on the other hand, is a soluble factor that provokes oxidative stress, loss of survivin and enhanced apoptosis in gastric cells (Valenzuela et al., 2013). In addition, *H. pylori* secreted peptidyl prolyl cis, trans-isomerase (HP0175) causes a gastric T-cell (Th17) response in patients with GC; leading to the production of IL-17 and IL-21, matrix degradation and the induction of pro-angiogenic pathways in response to HP0175 (Amedei et al., 2014).

The balance between these contradictory responses seems to be determined largely by intrinsic host-specific mechanisms. In agreement with this concept, knock-out animal models have revealed the importance of some host factors in modulating inflammation. For instance, TLR9 signaling has demonstrated anti-inflammatory effects by controlling exacerbated IL-17 production (Varga et al., 2016). Alternatively, the trefoil factor 1 suppresses *H. pylori*-induced inflammation by antagonizing NF-κB activation (Soutto et al., 2015). The NF-κB protein is a master regulator of the inflammatory transcriptional response to bacterial molecules, mainly in response to injected factors, such as peptidoglycan and CagA (Sokolova and Naumann, 2017). Importantly, certain NF-κB target genes such as TNFα, IL-1β, IL-2, and IL-8, present polymorphisms that are associated with increased risk for GC in patients (Valenzuela et al., 2015; Melchiades et al., 2017). However, it is important to consider the participation of other cell-signaling pathways in the production of inflammatory molecules. For instance, activation of the epidermal growth factor receptor (EGFR) is also associated with inflammation, DNA damage and gastric carcinogenesis (Sierra et al., 2017).

## *H. pylori* and oxidative/nitrosative stress

Oxidative stress in the gastric mucosa as a consequence of *H. pylori* infection is a crucial contributing factor to gastric carcinogenesis. Reportedly, infection was shown to correlate with increased damage due to oxidative stress of the gastric mucosa (Butcher et al., 2017). Although reversible upon bacterial eradication (Pignatelli et al., 2001), the consequences of oxidative stress are evident through observed changes in global lipid and protein expression (Baek et al., 2004) and an increase in damaged biomolecules, such as DNA (modification of bases, telomere shortening) (Obst et al., 2000; Lee et al., 2016). Additionally, anti-oxidant capacity is also reduced due to decreased levels of antioxidant molecules, such a glutathione (GSH) (Shirin et al., 2001) in the gastric mucosa of *H. pylori*-infected patients. The sources of reactive oxygen and nitrogen species are mainly neutrophils, macrophages and the gastric cells themselves (Valenzuela et al., 2015). Thus, in addition to the inflammatory cells, gastric cells produce ROS in response to *H. pylori* infection in different ways. For instance, by inducing pro-oxidant activities, such as the host spermine oxidase enzyme, which also promotes GC risk associated with *H. pylori* CagA cytotoxin activity (Chaturvedi et al., 2011), nicotinamide adenine dinucleotide phosphate (NADPH) oxidase (Cha et al., 2010) or generating ROS from mitochondria following activation of TLR4 signaling (Yuan et al., 2013). Also, *H. pylori*-neutrophil-activating protein A (NapA) activity promotes host gastric cell production of reactive oxygen intermediates (Wang et al., 2006b). Furthermore, the secreted bacterial GGT has emerged as a relevant pathogenic factor associated with oxidative stress and inflammation (Gong et al., 2010). GGT activity is associated with oxidation of membrane lipids using GSH as a substrate in the presence of iron (Valenzuela et al., 2015). Nitric oxide generated by the activity of iNOS in macrophages, lymphocytes and gastric cells of the infected mucosa yields highly toxic peroxynitrite, which damages proteins and DNA by generating nitrotyrosine and DNA adducts, respectively (Sakaguchi et al., 1999; Cherdantseva et al., 2014; Valenzuela et al., 2015).

On the other hand, gastric cells can escape from the oxidative stress induced by *H. pylori* infection by producing scavenger molecules, such as metallothioneins, which have been shown to be crucial factors in protecting against *H. pylori*-induced gastric erosive lesions in an animal model (Mita et al., 2008). Other relevant antioxidant mechanisms include those involving global regulation of energy metabolism, such as the AMP-activated protein kinase (AMPK) (Zhao et al., 2015) or the cyto-protective activity of the nuclear factor (erythroid-derived 2)-like 2 (Nrf2) (Yanaka, 2011). Such a harmful environment may represent major challenges for infecting bacteria, comparable even to that of acid stress. However, *H. pylori* is a well-adapted bacteria, able to resist oxidative stress due to mechanisms that permit the successful colonization and persistence in the gastric niche (Wang et al., 2006a). For instance, isogenic mutants deficient in superoxide dismutase (Seyler et al., 2001), NADPH quinone reductase (Wang and Maier, 2004), thioredoxin (Windle et al., 2000), and catalase (KatA) (Harris et al., 2003) activities are sensitive to oxidative stress and are largely defective in host colonization (Seyler et al., 2001). Furthermore, thioredoxin is an arginase chaperone and guardian against oxidative and nitrosative stress (McGee et al., 2006). Interestingly, the bacteria itself reportedly also produces ROS (Ding et al., 2007).

Table 1 summarizes the adaptive mechanisms due to *H. pylori* infection that could promote the acquisition of potentially malignant characteristics and favor disease progression to GC.

**Table 1:** Summary of literature references linking *H. pylori* infection to induction of the UPR, ER stress, apoptosis, autophagy, inflammation, and oxidative stress.

**Adaptive mechanism**	**Marker(s)**	**Tissue/cell type**	**References**
UPR–ER stress	BiP, XBP-1s, CHOP	Human and mouse models of gastric cancer Human-derived gastric cells/*H. pylori*	Baird et al., 2013
ER stress–Apoptosis	PERK, eIF2α, CHOP, BH3-only protein Bim, Bax	Human-derived gastric cells/*H. pylori* (VacA)	Akazawa et al., 2013
	Bax, cytochrome C release, CHOP, BiP	Murine and human gastric dendritic cells/ *H. pylori* (VacA)	Kim et al., 2015
UPR–ER stress–Autophagy	ULK1, ATG5, Beclin1, conversion LC3I to LC3II, PERK, CHOP, ATF4	Human-derived gastric cells/*H. pylori* HP0175	Halder et al., 2015
Apoptosis	bcl-xl, bcl-2, survivin	Human gastric biopsies Human-derived gastric cells/*H. pylori*	Valenzuela et al., 2010
Autophagy	Autophagosome detection, conversion LC3I to LC3II, GFP-LC3 detection, Atg5, Atg12	Human-derived gastric cells/*H. pylori* (± VacA)	Terebiznik et al., 2009
	p62, GFP-LC3 detection, conversion LC3I to LC3II, analysis genotypes of ATG16L1	Human gastric tissue Peripheral blood monocytes Primary gastric cells from mice/*H. pylori* (± VacA) Human-derived gastric cells/*H. pylori* (± VacA)	Raju et al., 2012
	*H. pylori*-related gene expression and associated polymorphisms	Human-derived gastric cells and macrophages (THP-1) /*H. pylori*	Castaño-Rodríguez et al., 2015
	Methylation status/expression of Atg genes, MAP1LC3Av1 methylation silencing, map1lc3a knock-down	Human gastric mucosa Rat gastric epithelial cells	Muhammad et al., 2017
Inflammation	iNOS, nitrotyrosine	iNOS deficient mice/*H. pylori* infection	Nam et al., 2004
	E-cad promoter methylation status, iNOS, NF-κB, nitric oxide production, DNA methyltransferase activity	Human gastric cancer cell lines (± IL-1β or *H. pylori*)	Huang et al., 2012
	Genomic DNA and methylation-specific analysis	Human non-cancerous corpus gastric mucosa (*H. pylori*)	Kaise et al., 2008
	Methylation analysis	Human gastric biopsies	Perri et al., 2007
	DNA methylation levels of six CpG islands; global DNA methylation levels	Mongolian gerbil (± 5-aza-20-deoxycytidine/*H. pylori*)	Niwa et al., 2013
	*H. pylori* colonization analysis	Mice were infected with *H. pylori*	Every et al., 2011
	TLR9 activation	Tlr9/IL-17-deficient mice/*H. pylori* cag T4SS	Varga et al., 2016
	TFF1, NF-κB-p65 nuclear staining, TNFα, IL-1β, chemokine [C-X-C motif] ligand 5, IL-4 receptor	Tff1-knockout mice/*H. pylori*	Soutto et al., 2015
	IL-2, polymorphism analysis	Human gastric biopsies	Melchiades et al., 2017
	EGFR, Cxcl1, Cxcl2, MAPK1/3, activator protein 1	Egfr-knockout mice/*H. pylori*	Sierra et al., 2017
	*H. pylori* HP0175	Non-ulcer dyspeptic patients	Oghalaie et al., 2016
Inflammation–Apoptosis	Ubiquitin-editing enzyme A20, NF-κB	Human-derived gastric cells/*H. pylori*	Lim et al., 2017
Oxidative stress–Apoptosis	Survivin	Human-derived gastric cells/*H. pylori* (± GGT)	Valenzuela et al., 2013
	Inflammatory cytokines, H_2_O_2_, antioxidants, apoptosis	Human-derived gastric cells/*H. pylori*	Ding et al., 2007
Inflammation–ROS	Translocation of HSP90β, Rac1 activation.	Human-derived gastric cells/*H. pylori*	Cha et al., 2010
Oxidative stress	iNOS, nitrotyrosine, 8-OH-dG	Human gastric biopsies	Pignatelli et al., 2001
	Proteomic analysis	Human gastric mucosa	Baek et al., 2004
	ROS, GSH, DNA fragmentation	Human-derived gastric cells/*H. pylori*	Obst et al., 2000
	NF-κB, SOD, PARP-1, γ-H2AX	Human gastric biopsies	Lee et al., 2016
	Chemiluminescence, thiobarbituric acid-reactive substance-equivalent levels, GSH	Human gastric biopsies	Jung et al., 2001
	GSH	Human gastric biopsies	Shirin et al., 2001
	Spermine oxidase, 8-OH-dG, apoptosis levels	Human-derived gastric cells/*H. pylori* (± CagA)	Chaturvedi et al., 2011
	TLR4, ROS	Human gastric biopsies Human-derived gastric cells	Yuan et al., 2013
	*H. pylori*-NapA	C57BL/6J mice *H. pylori* (± NapA)	Wang et al., 2006b
	*H. pylori*-GGT, H_2_O_2_, IL-8, 8-OH-dG	Primary gastric epithelial cells Human-derived gastric cells	Gong et al., 2010
	Metallothionein, NF-κB	Metallothionein-null mice	Mita et al., 2008
	AMPK, compound 13	Primary gastric epithelial cells Human-derived gastric cells	Zhao et al., 2015
	SOD	*H. pylori*-sodB mutant C57BL/6J mice	Seyler et al., 2001
	NADPH quinone reductase	*H. pylori-*MdaB protein C57BL/6J mice	Wang and Maier, 2004
	Thioredoxin reductase	*H. pylori*-thioredoxin	Windle et al., 2000
	Catalase (KatA), KatA-associated protein (KapA)	C57/BL6 mice KatA/KapA-deficient-*H. pylori* mutants	Harris et al., 2003
	Thioredoxin 1	*H. pylori*-trxA1 mutant	McGee et al., 2006
Nitrosative stress	Peroxynitrite, iNOS, nitrotyrosine,	Human gastric biopsies	Sakaguchi et al., 1999
	iNOS	Human gastric biopsies	Cherdantseva et al., 2014

*XBP1s, spliced X-box binding protein 1; ULK1, serine/threonine-protein kinase ULK1; ATG5, autophagy protein 5; LC3, microtubule-associated protein 1A/1B-light chain 3; ATF4, activating transcription factor 4; iNOS, nitric oxide synthase; NF-κB, nuclear factor kappa-light-chain-enhancer of activated B cells; TFF1, trefoil factor 1; TNFα, tumor necrosis factor alpha; IL, interleukin; EGFR, epidermal growth factor receptor, MAPK, mitogen-activated protein kinase; H_2_O_2_, hydrogen peroxide; HSP90β, heat shock protein 90 beta; 8-OH-dG, 8-hydroxy-2'-deoxyguanosine; SOD, superoxide dismutase; PARP-1; poly [ADP-ribose] polymerase 1; NapA, neutrophil activating protein A; AMPK, AMP-activated protein kinase*.

## Discussion-potential therapeutic strategies

Understanding the regulation of adaptive chronic mechanisms by *H. pylori* may open up therapeutic options to prevent the progression of GC precursor lesions.

In line with this, PERK has emerged as potential therapeutic target in cancer. The compound GSK2606414 (GlaxoSmithKline®) was the first reported small molecular inhibitor of PERK, which is highly specific (Axten et al., 2012). When administered orally, GSK2606414 inhibits tumor growth in a dose-dependent manner in mice with human pancreatic BxPC3 tumors (Axten et al., 2012). Moreover, a second PERK inhibitor, GSK2656157, was developed for preclinical studies exhibiting promising results in xenograft mouse models bearing human tumors (Atkins et al., 2013; Axten et al., 2013). More recently, a new role for PERK signaling in promoting cancer cell migration and invasion has been proposed. Medulloblastoma cell lines treated with GSK2606414 exhibited a significant reduction in the level of phospho-eIF2α and concomitant suppression of cell migration and invasion (Jamison et al., 2015). Overall, these observations support the idea that, besides its classical role in UPR and apoptosis, PERK also promotes an aggressive cellular phenotype by increasing the migratory and invasive potential. With this in mind, it is not surprising that there is considerable interest in defining whether PERK contributes to tumor progression and whether it represents a suitable therapeutic target in cancer (Pytel et al., 2016).

On the other hand, cancer cells are thought to use autophagy as a source of energy in the unfavorable metastatic environment, and a number of clinical trials are now revealing the promising role of chloroquine, an anti-malaria drug and autophagy inhibitor, that also suppresses tumor growth and metastasis (Kimura et al., 2013; Maes et al., 2014). In addition, a potential role for gastrin in the regulation of autophagy has been recently proposed, opening up new avenues for the treatment of GC by targeting autophagy in combination with conventional cytostatic drugs (Rao et al., 2017). Furthermore, the use of statins has been reported to enhance *H. pylori* eradication by antibiotics and to reduce *H. pylori*-mediated inflammation by promoting autophagy (Liao et al., 2016); however, the mechanisms underlying these effects remain unclear.

Studies using pharmacological approaches to modulate inflammation or knock-out animal models of infection lacking cytokine receptors have highlighted the crucial importance of pro-inflammatory responses in promoting *H. pylori*-associated gastric carcinogenesis (Waghray et al., 2010; Huang et al., 2013; Oshima et al., 2014). This may point towards the importance of protective natural dietary compounds and micronutrients in reducing inflammation and preventing carcinogenesis (Wang, 2014; Noto and Peek, 2015; Zitvogel et al., 2017). Thus, natural anti-inflammatory compounds may hold considerable promise in preventing *H. pylori*-associated gastric pathologies (Wang, 2014).

In summary, the mechanisms described here may be exploited as pharmacological targets, in the perspective of opening up new therapeutic alternatives for non-invasive risk control in GC. This possibility is particularly intriguing when dealing with GC, which represents the leading cause of cancer deaths in several countries (IARC, 2014). Many highly specific inhibitors to regulate and/or control these adaptive mechanisms are already available and being evaluated in clinical trials for other cancers.

## Author contributions

PD, MV, and JB drafted the manuscript with support from AQ. PD drafted summary figure and table. PD, MV, JB, and AQ critically revised the article. All authors read and approved the final article.

### Conflict of interest statement

The authors declare that the research was conducted in the absence of any commercial or financial relationships that could be construed as a potential conflict of interest.
